# Preliminary report of Iranian Registry of Alzheimer's disease in Tehran province: A cross‐sectional study in Iran

**DOI:** 10.1002/hsr2.952

**Published:** 2022-11-22

**Authors:** Javad Fahanik‐Babaei, Mohsen Sedighi, Soraya Mehrabi, Omid Pournik, Abbas Sheikh Taheri, Leila Kamalzadeh, Mahsa Zarei, Mehrdad Roghani, Fereshteh Golab, Mostafa Almasi, Afshin Etezadi, Siamak Afshin‐Majd, Seyed Kazem Malakouti, Majid Rajabi, Mehdi Moghaddasi, Gholamreza Hajati, Fatemeh Golmohammadi Khamne, Arzhang Jafari, Alireza Amanollahi, Tourandokht Baluchnejadmojarad

**Affiliations:** ^1^ Registry Program of Cognitive Deficit and Alzheimer's Disease Information in Tehran Province Tehran Iran; ^2^ Electrophysiological Research Center Tehran University of Medical Sciences Tehran Iran; ^3^ Trauma and Injury Research Center Iran University of Medical Sciences Tehran Iran; ^4^ Department of Physiology, School of Medicine Iran University of Medical Sciences Tehran Iran; ^5^ Preventive Medicine and Public Health Research Center, Psychosocial Health Research Institute Iran University of Medical Sciences Tehran Iran; ^6^ School of Health Management and Information Sciences Iran University of Medical Sciences Tehran Iran; ^7^ Mental Health Research Center Iran University of Medical Sciences Tehran Iran; ^8^ School of Behavioral Sciences and Mental Health, Tehran Psychiatric Institute Iran University of Medical Sciences Tehran Iran; ^9^ Neurophysiology Research Center Shahed University Tehran Iran; ^10^ Cellular and Molecular Research Center Iran University of Medical Sciences Tehran Iran; ^11^ Faculty of Medicine Iran University of Medical Sciences Tehran Iran; ^12^ Iranian Dementia and Alzheimer's Association Tehran Iran; ^13^ Department of Biology, Faculty of Science Islamic Azad University of Shahr‐E‐Qods Tehran Iran; ^14^ Department of Epidemiology, School of Public Health and Safety Shahid Beheshti University of Medical Sciences Tehran Iran

**Keywords:** Alzheimer disease, data collection, disease management, registries

## Abstract

**Background and Aims:**

Alzheimer's disease (AD) is the main cause of dementia and over the 55 million people live with dementia worldwide. We aimed to establish the first database called the Iranian Alzheimer's Disease Registry to create a powerful source for future research in the country. In this report, the design and early results of the Iranian Alzheimer's Disease Registry will be described.

**Methods:**

We performed this multicenter investigation and patients' data including age, sex, educational level, disease status, Mini‐Mental State Examination (MMSE), and Geriatric Depression Scale (GDS) from 2018 to 2021 were collected, registered, and analyzed by GraphPad Prism software.

**Results:**

Totally 200 AD patients were registered in our database. 107 (54%) were women and age of 147 (74%) were over 65. The mean age for men and women was 76.20 ± 8.29 and 76.40 ± 8.83 years, respectively. 132 (66%) were married and 64 (32%) were illiterate. Also, 94 (47%) were in the moderate stage of disease, and 150 (75%) lived at home together with their families. The most frequent neurological comorbidity was psychosis (*n* = 72, 36%), while hypertension was the most common non‐neurological comorbidity (*n* = 104, 52%). The GDS score of women in the mild stage (5.23 ± 2.9 vs. 6.9 ± 2.6, *p* = 0.005) and moderate stage (5.36 ± 2.4 vs. 8.21 ± 2.06, *p* = <0.001) of the disease was significantly greater than men. In univariate analysis, MMSC score was remarkably associated with stroke (*β* = −2.25, *p* = 0.03), psychosis (*β* = −2.18, *p* = 0.009), diabetes (*β* = 3.6, p = <0.001), and hypercholesteremia (*β* = 1.67, *p* = 0.05). Also, the MMSE score showed a notable relationship with stroke (*β* = −2.13, *p* = 0.05) and diabetes (*β* = 3.26, *p* = <0.001) in multivariate analysis.

**Conclusion:**

Iranian Alzheimer's Disease Registry can provide epidemiological and clinical data to use for purposes such as enhancing the current AD management in clinical centers, filling the gaps in preventative care, and establishing effective monitoring and cure for the disease.

## 1 INTRODUCTION

Neurodegenerative diseases have been considered the major causes of disability and death in older people.^1^ Alzheimer's disease (AD), other dementias, and Parkinson's disease (PD) are the most frequent neurodegenerative disorders and the debilitating nature of neurodegeneration has made it a major cause of dependence and disability across the world.^2^ Based on the Global Burden of Disease (GBD) 2016 report, the age‐standardized rate of prevalence for AD and other dementias and PD in the Eastern Mediterranean Region (EMR) was estimated 759.8 and 87.1 per 100,000 people, respectively.^3^

Dementia is defined as a decline in cognitive function impairing a person's previous status of social and occupational function. AD is the most common type of dementia, whereas vascular dementia is the second. Other major types of diseases causing dementia are psychiatric disorders, frontotemporal dementia, and Lewy body dementia.^4^ AD imposes a negative impact not only on the patients but also on their relatives, particularly those directly responsible for their care. A recent report from the World Health Organization (WHO) showed that neurological disorders, ranging from epilepsy to AD, traumatic brain injury (TBI), multiple sclerosis (MS), neuroinfections, stroke, and PD, affect up to one billion people across the world and dementia has been considered as one of the neurological diseases that encourages governments to provide support for caregivers.^5^ Global estimated prevalence for dementia is 3.9% for people over 60, with the local prevalence being 4.0% in China and Western Pacific regions, 1.6% in Africa, 4.6% in Latin America, 6.4% in North America, and 5.4% in Western Europe.^6^

World Alzheimer Report in 2021 released by Alzheimer Disease International (ADI) reported that over 55 million dementia people live in the world. This number will go up with an aging population to more than 78 million by 2030 in the world.^7^ World Alzheimer Report has shown that major barriers to AD and dementia diagnosis in people and caregivers include lack of access to experienced clinicians (47%), fear of diagnosis (46%), and cost (34%).^8^ Most of the people with AD and their family carers feel isolated in society and lose their friends and even family members.^9^

Surgeon General of the US Public Health Service established the National Committee on Vital and Health Statistics (NCVHS) in 1949.^10^ NCVHS introduced registries as an organized system for the gathering, storage, retrieval, processing, and spread of data to individuals with certain disorders or conditions that put them at risk of a health‐related event or adverse health effects.^11^ Registry of Patient Registries (RoPR) is a central listing of patient registries developed by the Agency for Healthcare Research and Quality (AHRQ) in 2012 in collaboration with the National Library of Medicine. The RoPR was introduced to enhance transparency and reduce redundancy in registry‐based research.^12^ Dementia registry systems cluster in Western Europe (mainly in Scandinavia and the United Kingodm), North America (mainly the United States), and only three active registries in other countries including Argentina, Colombia, and South Korea.^13^

Patient registry systems are a valuable source to help address important challenges in research, care, and policy. Registries, well embedded in numerous fields of public health and medicine, are new in dementia, relatively.^14^ Hence, the Iranian Alzheimer's disease Registry project was designed and developed as the first registry database for patients with AD in Iran. The objectives of the Iranian Alzheimer's disease Registry are as follows: (1) to determine the incidence of AD in the province of Tehran, (2) to define the known risk factors of AD, (3) to improve the quality of care, and (4) to identify problems related to the further national AD registrations. This paper describes the preliminary report of the Iranian Alzheimer's disease Registry project.

## 2 MATERIALS AND METHODS

### 2.1 Study design and ethics

Iranian Alzheimer's disease Registry is a registry of data on AD patients from outpatient neurology clinics and hospitals 1 in Tehran city of Iran with more than 13 million members of the general population. Iranian Alzheimer's disease Registry was designed and approved by the Medical Research Ethics Committee (R.IUMS.REC.1396.32623) and informed consent was obtained from the patients participating in the study. Data were collected from these centers between September 2018 and December 2021. Subjects were entered into this investigation if they were diagnosed with AD by a neurologist and/or psychiatrist based on cognitive tests and clinical examination.

### 2.2 Clinical assessment of AD

Experienced neurologists and neuropsychiatrists examined all patients through past medical history, clinical examination, neuropsychological evaluation, laboratory tests, and neuroimaging. Also, further assessment was performed to evaluate memory deficit and other thinking abilities, quality of the judge, and identify changes in behavior to determine stages of AD and comorbidities based on the International Classification of Diseases, 10th Revision (ICD‐10).^15^ AD is classified into mild, moderate, and severe stages in terms of Clinical Dementia Rating (CDR) which is a global dementia rating scale to evaluate cognitive change, identify the presence of dementia, and determine severity of dementia from very mild (CDR 0.5) to mild (CDR 1), moderate (CDR 2), and severe (CDR 3).^16^ In mild disease, patients experience some functional dependence, such as trouble managing finances. In the moderate form of the disease, patients are more dependent on others, have difficulty with bathing and shopping, and often are not able to drive. The severe form is characterized by total dependence on care providers and deficit in motor and balance.^17^

### 2.3 Assessment of comorbidities

Neurological comorbidities were investigated through brain magnetic resonance imaging (MRI) and computed tomography (CT) to detect brain ischemia due to transient ischemic attack or mild ischemic stroke and small vessel disease.^18^ Also, PD was diagnosed in the case of micrographia, visible resting tremor, hypomimia, slowed repetitive movements, impaired arm swing, and pain in muscles.^19^ Patients with frequent extrapyramidal signs, more rapid cognitive dysfunction, and greater deficit in the putative neuropsychological examination of frontal lobe function were assessed for existing depression and psychosis as psychotic symptoms of AD.^20^

For none neurological comorbidities, patients were considered diabetics if they had hemoglobin A1c ≥ 6.5%, nonfasting blood glucose ≥ 200 mg/dl, and fasting blood glucose ≥ 126 mg/dl.^21^ Hypertension in patients was diagnosed if blood pressure was consistently above130 and/or above 80 mmHg^22^ and hypercholesterolemia was defined as serum total blood cholesterol of 200 mg/dl or more, according to the National Cholesterol Education Program (NCEP) III guidelines.^23^ Diagnosis of cardiovascular disease (CVD) was made through extraction of all ICD codes for atherosclerosis, claudication, angina pectoris, myocardial infarction (MI), ischemic heart disease (IHD), coronary artery bypass graft (CABG) surgery, and percutaneous coronary intervention (PCI).^24^

### 2.4 Neuropsychological assessment

Mini‐Mental State Examination (MMSE) is a validated tool for assessing cognition in the clinical setting.^25^ The Farsi version of MMSE (F‐MMSE) was validated by Seyedian et al.^26^ that consists of an 11‐item measure to test five aspects of cognitive function including orientation, registration, attention and calculation, recall, and language. 30 score considers the maximum and a cut‐off point of below 22 shows a definite cognitive deficit.

Geriatric Depression Scale (GDS) instrument is widely used for evaluating depression in later life. A short form of the GDS with 15 questions is available to use in clinical practice more attractively, as it can considerably lower administration time.^27^ We used the Persian version of GDS developed by Malakouti et al.^28^ to reach the medical diagnosis of a major depressive episode in older people.

### 2.5 Data collection

A questionnaire was designed by neurologists and psychiatrists and validated by the institutional quality control committee experts. The questionnaire is composed of multiple different parts to collect demographic information (age, sex, marriage, family history, education, and occupation), current clinical presentations to the clinic or medical center (stage of the AD, neurological comorbidities, non‐neurological comorbidities, cigarette consumption, and status of care), paraclinical data (brain images, laboratory tests) MMSE, and GDS.

### 2.6 Statistical analysis

Patients' data was stored in a database, namely Iranian collaborated AD, and then were transferred to the Iranian Alzheimer's Disease Registry. Identified AD cases were registered in the database and patient data were subsequently processed and analyzed using GraphPad Prism 8.1.1 (GraphPad). Categorical outcomes were shown as frequency and continuous outcomes were expressed as the mean ± standard deviation (SD). To compare continuous outcomes in patients, an independent *t*‐test was used and linear regression analysis (univariate, multivariate) was performed to explore the association between MMSE and GDS score with patient characteristics. *p* Value was set as ≤0.05.

## 3 RESULTS

From 2018 to 2020, a total of 200 AD patients were enrolled in the database, of whom 107 (54%) were women. 147 (74%) were older than 65 years and the mean age for men and women was 76.20 ± 8.29 and 76.40 ± 8.83 years, respectively. Among the 200 patients with AD, 131 (67%) had a family history of AD in first degree relatives, 132 (66%) were married, and 103 (51%) had more than three children. Regarding education and occupation, 64 (32%) were illiterate, 91 (45%) women were homemaker, and 42 (21%) men were worker (Table 1).

**Table 1:** Sociodemographic characteristics of the participants (*n* = 200)

**Variables** a	**Number**	**Percentage**
Age of the patients		
25–44 (young age)	0	0
45–64 (middle age)	53	26
65–98 (older age)	147	74
Family history of AD		
First‐degree relatives	131	67
Second‐degree relatives	44	22
Third‐degree relatives	12	5
Unknown	13	6
Gender		
Men	93	46
Women	107	54
Marital status		
Single	2	1
Married	132	66
Divorced	2	1
Widow	64	32
Number of children		
0	5	3
1	8	4
2	24	12
3	60	30
>3	103	51
Educational level		
Illiterate	64	32
Primary school	56	28
High school	59	30
University degree	21	10
Occupation		
Homemaker (women)	91	45
Employee (men/women)	39/11	20/5
Worker (men)	42	21
Retired (men/women)	12/5	6/3

*Note*: First‐degree relative (parents), second‐degree relative (brother/sister), third‐degree relative (uncle/aunt), employee (a person who is employed by the government), worker (a person who is employed by a private company).

Abbreviation: AD, Alzheimer's disease.

^a^ Categorical data are presented as frequency (%).

**Figure 1:**
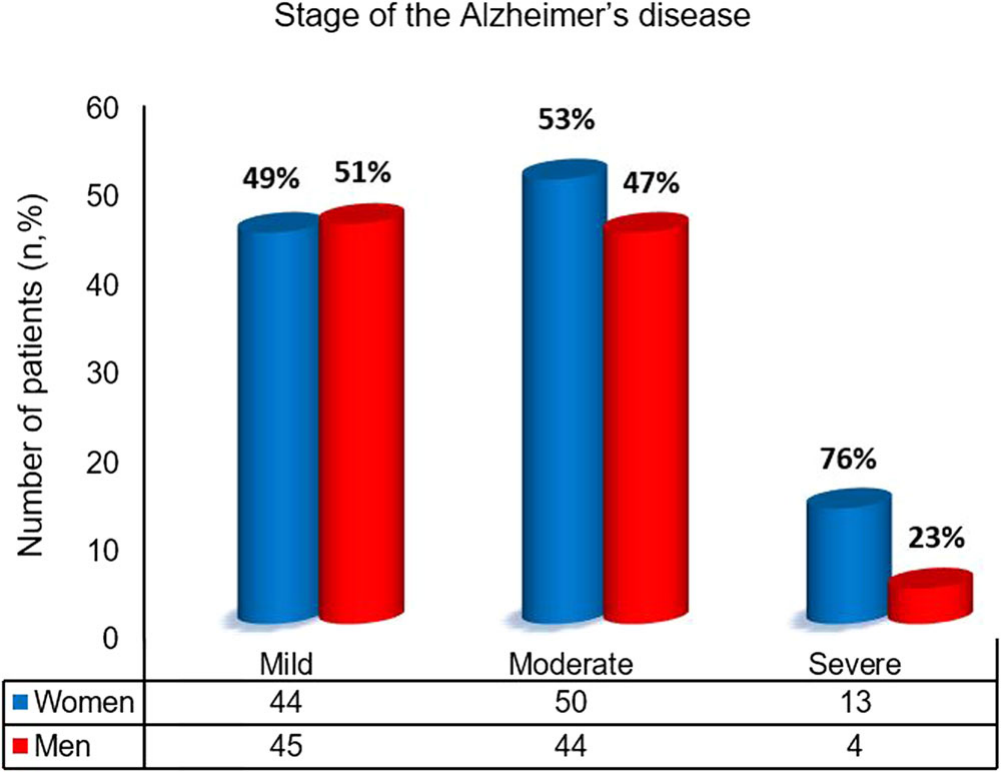
Distribution of men and women in different stages of Alzheimer's disease. Categorical data are presented as frequency (%).

Table 2 shows that 94 (47%) were in the moderate stage of AD and 68 (34%) experienced depression. Also, 36 (18%) were heavy smoker (>10 cigarettes per day).^29^ Of all the none neurological diseases investigated among AD patients, hypertension in 104 (52%) and hypercholesterolemia in 80 (40%) were the most common. Of the 200 participants, 150 (75%) lived at home and received care from their families. Also, 44 (22%) showed symptoms of severe AD and dementia. Distribution of men and women in different stages of AD is illustrated in Figure 1, indicating that the severe stage of disease was more evident in women than men (76% vs. 23%).

**Table 2:** Baseline characteristics of the participants (*n* = 200)

**Variables** a	**Number**	**Percentage**
Stage of disease		
Mild	89	45
Moderate	94	47
Severe	17	8
Neurological comorbidities		
Stroke	35	18
Parkinson disease	6	3
Depression	68	34
Psychosis	72	36
Non‐neurologic comorbidities		
Diabetes	53	27
Hypertension	104	52
Hypercholesteremia	80	40
Cardiovascular disease	62	31
Smoking		
None	160	80
Light smoker (<10 CPD)	4	2
Heavy smoker (>10 CPD)	36	18
Patient care status		
Independent living	30	15
Family living	150	75
Home health care	17	9
Adult daycare	3	1
AD Dementia		
Yes	44	22
No	64	32
Undiagnosed	8	4

Abbreviations: AD, Alzheimer's disease; CPD, cigarettes per day.

^a^ Categorical data are presented as frequency (%).

Regarding the assessment of cognitive status, there was no statistically notable difference between men and women for MMSE score in different stages of the disease, nevertheless, women got significantly higher GDS scores than men in the mild (5.23 ± 2.9 vs. 6.9 ± 2.6, *p* = 0.005) and moderate stage (5.36 ± 2.4 vs. 8.21 ± 2.06, *p* = <0.001) of the AD. Furthermore, the MMSE score in women showed a significant decrease (21.33 ± 3.5 vs. 19.03 ± 4.6, *p* = 0.008) when compared between mild and moderate stage of AD, while the GDS score showed a significant increase (6.9 ± 2.6 vs. 8.21 ± 2.06, *p* = 0.003) (Table 3). As described in Table 4, univariate analysis revealed a significant association between MMSE score and stroke (*β* = −2.25, *p* = 0.03), psychosis (*β* = −2.18, *p* = 0.009), diabetes (*β* = 3.6, *p* = <0.001), and hypercholesteremia (*β* = 1.67, *p* = 0.050). In multivariate analysis, we observed a significant relationship between MMSE score and stroke (*β* = −2.13, *p* = 0.05) and diabetes (*β* = 3.26, *p* = <0.001).

**Table 3:** MMSE and GDS score of patients in different stages of Alzheimer's disease

Stage of disease	Mild (A) (*n* = 89)	*p* Value	Moderate (B) (*n* = 94)	*p* Value	Severe (C) (*n* = 17)	*p* Value	A versus B ***p* Value**
**Cognitive test** a							
MMSE score							
Men	20.32 ± 5.3	0.30	18.82 ± 3.2	0.80	15 ± 5.1	‐	0.10
Women	21.33 ± 3.5		19.03 ± 4.6		15 ± 5.4		0.008
GDS score							
Men	5.23 ± 2.9	0.005	5.36 ± 2.4	<0.001	NA	‐	0.80
Women	6.9 ± 2.6		8.21 ± 2.06		NA		0.003

Abbreviations: GDS, geriatric depression scale; MMSE, Mini‐Mental State Examination.

a Continues data are presented as mean ± standard deviation and analyzed by independent student *t*‐test. Cognitive status was defined using the patients' MMSE scores (0–9 = *severe*; 10–19 = *moderate*; 20–26 = *mild*; +27 = *normal*) and GDS score (0–4 = *normal*; 5–8 = *mild depression*; 9–11 = moderate depression; 12–15 = severe depression). *p* ≤ 0.05 is significant.

**Table 4:** Association between MMSE and GDS score with characteristics of Alzheimer's disease patients

Cognitive assessment	Patients' variables	Univariate		Multivariate	
		Coefficient	*p* Value	Coefficient	*p* Value
MMSE score	Age	−0.008	0.87	−0.003	0.94
	Gender	0.014	0.98	1.33	0.87
	Smoking	1.46	0.19	1.64	0.13
	Stroke	−2.25	0.03	−2.13	0.05
	Parkinson disease	1.03	0.63	0.94	0.64
	Depression	1.37	0.11	0.87	0.23
	Psychosis	−2.18	0.009	−1.47	0.07
	Diabetes	3.6	<0.001	3.26	<0.001
	Hypercholesteremia	1.67	0.05	0.57	0.53
	Hypertension	0.55	0.52	−0.091	0.92
	Cardiovascular disease	0.89	0.32	1.06	0.24
GDS score	Age	−0.03	0.45	−0.04	0.38
	Gender	−0.21	0.80	−0.60	0.45
	Smoking	0.97	0.32	1.03	0.32
	Stroke	0.61	0.60	2.02	0.14
	Parkinson disease	4.61	0.13	6.05	0.65
	Depression	0.62	0.42	0.68	0.40
	Psychosis	−0.89	0.30	−1.52	0.11
	Diabetes	−0.52	0.52	0.05	0.96
	Hypercholesteremia	−0.22	0.78	0.39	0.65
	Hypertension	−0.94	0.22	−1.71	0.07
	Cardiovascular disease	−0.32	0.70	0.06	0.95

*Note*: Univariate and multivariate analysis of cognitive assessment test and patients' variables. *p* ≤ 0.05 is significant.

Abbreviations: GDS, geriatric depression scale; MMSE, Mini‐Mental State Examination.

## 4 DISCUSSION

The most prevalent form of dementia is AD which may account for 60%–70% of dementia subjects.^30^ AD is commonly described as a progressive neurodegenerative disease typified by gradual memory decline, cognitive impairments, difficulty in navigating, and even executive function.^31^ These neuropsychiatric symptoms, which are categorized as behavioral and psychological symptoms of dementia (BPSD), are prevalent and occur during the gradual progress of the disease.^32^ In the first Iranian Alzheimer's Disease Registry, which was initiated in the province of Tehran in 2018, 45% of AD patients were in the mild stage of disease, 47% were moderate, and 8% were in the late stage of AD. Moreover, MMSE and GDS scores in women differed significantly between mild and moderate stage of AD. Also, the MMSE score was associated with neurological and none neurological comorbidities in AD patients. The gender‐specific difference in the phenotype of AD is to be explained by differences in brain morphology and function as well as higher susceptibility for pathological lesions in women and also greater cognitive reserve in men. Sex differences have also been reported for the expression of antioxidative enzymes and post‐menopausal hormonal changes.^33^

Patient registries are strong tools to monitor the course of diseases, prevent and treat the diseases, and evaluate parameters that influence prognosis and quality of life.^34^ Patient registries provide a comprehensive database, and therefore, generated data may be generalizable to a variety of patients.^35^ The Iranian Alzheimer's Disease Registry generated information about patients with AD who were visited and consulted by neurologists and psychiatrists in outpatient clinics and medical centers for 2 years. Our population contained more women than men (107 vs. 93) which is in parallel with other databases in the literature^15^ and indicates a higher incidence of AD in women than in men.^36^, ^37^, ^38^

ADI report calculates that globally 75% of dementia people are misdiagnosed and this figure may be as high as 90% in some low‐ and middle‐income regions of the world, where lack of awareness of dementia remains a major barrier to AD diagnosis.^7^ The World Alzheimer Report 2010 reported that the economic cost of dementia was USD 604 billion which was equal to 1% of the global gross domestic product.^39^ Based on the ADI report, this figure showed an increase of 35% in 2015 and it is predicted that the cost of care will respectively grow to 1 and 2 trillion USD by 2018 and 2030.^40^ The annual cost of care for mild, moderate, and severe AD subjects in Iran was estimated to be 434D, 1313, and 2480 USD, respectively. These figures in Iran are lower when compare to the average dementia cost in regions with upper‐middle‐income.^41^ The current report is the first Iranian Alzheimer's database provided by our institute in collaboration with the Ministry of Health and Education. Prior articles by other researchers in other countries reported that a commission is needed in all countries for nationally representative surveys that are repeated regularly to follow trends in AD and dementia. Also, demographic factors including age, gender, and educational level are important elements that should be considered for evaluation.^42^, ^43^

To the best of our knowledge, the Iranian Alzheimer's Disease Registry is the first of its kind for patients with AD in Iran. However, our program has some limitations. First, the number of patients included in our primary report was small and some of the outcomes of the current investigation such as prevalence of AD cannot be generalized to the general Iranian population. Second, some barriers such as lack of a suitable infrastructure for implementation and limited centers and clinics to participate without expecting financial supports restrict our efforts for continuing patient registration.

In conclusion, the findings from the present study showed a broader spectrum of behavioral symptoms attributed to dementia with a predominance of depression in women. Also, metabolic and neurological comorbidities affected remarkably MMSE score in AD patients. Iranian Alzheimer's Disease Registry can provide epidemiological and clinical data to use for purposes such as enhancing the current AD management in clinical centers, filling the gaps in preventative care, establishing effective monitoring and cure for the disease, and even local and international investigations.

## AUTHOR CONTRIBUTIONS

**Javad Fahanik‐Babaei**: Conceptualization; formal analysis; writing – original draft; writing – review and editing. **Mohsen Sedighi**: Conceptualization; formal analysis; investigation; software; writing – original draft; writing – review and editing. **Soraya Mehrabi**: Formal analysis; investigation; methodology; writing – original draft; writing – review and editing. **Omid Pournik**: Data curation; investigation; methodology; software. **Abbas Sheikh Taheri**: data curation; formal analysis; investigation; software. **Leila Kamalzadeh**: Investigation; methodology. **Mahsa Zarei**: Formal analysis; investigation; methodology. **Mehrdad Roghani**: Formal analysis; investigation; writing – original draft. **Fereshteh Golab**: Formal analysis; investigation. **Mostafa Almasi**: Investigation; methodology. **Afshin Etezadi**: Formal analysis; investigation. **Siamak Afshin‐Majd**: Formal analysis; investigation; methodology. **Seyed Kazem Malakouti**: Data curation; formal analysis; investigation. **Majid Rajabi**: Investigation; methodology. **Mehdi Moghaddasi**: Funding acquisition; investigation. **Gholamreza Hajati**: Investigation; methodology. **Fatemeh Golmohammadi Khamne**: Formal analysis; investigation. **Arzhang Jafari**: Formal analysis; investigation. **Alireza Amanollahi**: Conceptualization; formal analysis; investigation; writing – review and editing. **Tourandokht Baluchnejadmojarad**: Conceptualization; methodology; project administration; supervision; writing – original draft; writing – review and editing.

## CONFLICT OF INTEREST

The authors declare no conflict of interest.

## TRANSPARENCY STATEMENT

The lead author Mohsen Sedighi, Tourandokht Baluchnejadmojarad affirms that this manuscript is an honest, accurate, and transparent account of the study being reported; that no important aspects of the study have been omitted; and that any discrepancies from the study as planned (and, if relevant, registered) have been explained.

## Supporting information

Supporting information.

Supporting information.

## Data Availability

The data that support the findings of this study are available from the corresponding author upon reasonable request.

## ENDNOTE

1 Psychosocial Health Research Institute, Mental Health Research Center, Firoozgar Hospital, Rasoul Akram Hosital, Tehran Psychiatric Institute, Mostafa Khomeini Hospital.
