# Supplementary material for: Preliminary report of Iranian Registry of Alzheimer's disease in Tehran province: A cross‐sectional study in Iran
Source: Health Sci Rep. 2022 Nov 22;5(6):e952. doi: 10.1002/hsr2.952 (PMC9682202; doi:10.1002/hsr2.952)
Supplement: Supplementary file 1 — Supporting information. [file HSR2-5-e952-s002.pdf]

Mini-Mental State Examination (MMSE)

Patient's Name: ________________________________ Date: ____________

<u>Instructions:</u> Ask the questions in the order listed. Score one point for each correct response within each question or activity.

Maximum Score	Patient's Score	Questions
5		"What is the year? Season? Date? Day of the week? Month?"
5		"Where are we now: State? County? Town/city? Hospital? Floor?"
3		The examiner names three unrelated objects clearly and slowly, then asks the patient to name all three of them. The patient's response is used for scoring. The examiner repeats them until patient learns all of them, if possible. Number of trials: ___________
5		"I would like you to count backward from 100 by sevens." (93, 86, 79, 72, 65, …) Stop after five answers.<br>Alternative: "Spell WORLD backwards." (D-L-R-O-W)
3		"Earlier I told you the names of three things. Can you tell me what those were?"
2		Show the patient two simple objects, such as a wristwatch and a pencil, and ask the patient to name them.
1		"Repeat the phrase: 'No ifs, ands, or buts.'"
3		"Take the paper in your right hand, fold it in half, and put it on the floor." (The examiner gives the patient a piece of blank paper.)
1		"Please read this and do what it says." (Written instruction is "Close your eyes.")
1		"Make up and write a sentence about anything." (This sentence must contain a noun and a verb.)
1		"Please copy this picture." (The examiner gives the patient a blank piece of paper and asks him/her to draw the symbol below. All 10 angles must be present and two must intersect.)<br>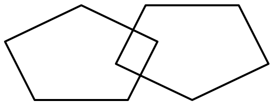
30		TOTAL

(Adapted from Rovner & Folstein, 1987)

Instructions for administration and scoring of the MMSE

Orientation (10 points):

- Ask for the date. Then specifically ask for parts omitted (e.g., "Can you also tell me what season it is?"). One point for each correct answer.
- Ask in turn, "Can you tell me the name of this hospital (town, county, etc.)?" One point for each correct answer.

Registration (3 points):

- Say the names of three unrelated objects clearly and slowly, allowing approximately one second for each. After you have said all three, ask the patient to repeat them. The number of objects the patient names correctly upon the first repetition determines the score (0-3). If the patient does not repeat all three objects the first time, continue saying the names until the patient is able to repeat all three items, up to six trials. Record the number of trials it takes for the patient to learn the words. If the patient does not eventually learn all three, recall cannot be meaningfully tested.
- After completing this task, tell the patient, "Try to remember the words, as I will ask for them in a little while."

Attention and Calculation (5 points):

- Ask the patient to begin with 100 and count backward by sevens. Stop after five subtractions (93, 86, 79, 72, 65). Score the total number of correct answers.
- If the patient cannot or will not perform the subtraction task, ask the patient to spell the word "world" backwards. The score is the number of letters in correct order (e.g., dlrow=5, dlorw=3).

Recall (3 points):

- Ask the patient if he or she can recall the three words you previously asked him or her to remember. Score the total number of correct answers (0-3).

Language and Praxis (9 points):

- Naming: Show the patient a wrist watch and ask the patient what it is. Repeat with a pencil. Score one point for each correct naming (0-2).
- Repetition: Ask the patient to repeat the sentence after you ("No ifs, ands, or buts."). Allow only one trial. Score 0 or 1.
- 3-Stage Command: Give the patient a piece of blank paper and say, "Take this paper in your right hand, fold it in half, and put it on the floor." Score one point for each part of the command correctly executed.
- Reading: On a blank piece of paper print the sentence, "Close your eyes," in letters large enough for the patient to see clearly. Ask the patient to read the sentence and do what it says. Score one point only if the patient actually closes his or her eyes. This is not a test of memory, so you may prompt the patient to "do what it says" after the patient reads the sentence.
- Writing: Give the patient a blank piece of paper and ask him or her to write a sentence for you. Do not dictate a sentence; it should be written spontaneously. The sentence must contain a subject and a verb and make sense. Correct grammar and punctuation are not necessary.
- Copying: Show the patient the picture of two intersecting pentagons and ask the patient to copy the figure exactly as it is. All ten angles must be present and two must intersect to score one point. Ignore tremor and rotation.

(Folstein, Folstein & McHugh, 1975)

Interpretation of the MMSE

Method	Score	Interpretation
Single Cutoff	<24	Abnormal
Range	<21	Increased odds of dementia
	>25	Decreased odds of dementia
Education	21	Abnormal for 8th grade education
	<23	Abnormal for high school education
	<24	Abnormal for college education
Severity	24-30	No cognitive impairment
	18-23	Mild cognitive impairment
	0-17	Severe cognitive impairment

Sources:

- Crum RM, Anthony JC, Bassett SS, Folstein MF. Population-based norms for the mini-mental state examination by age and educational level. *JAMA*. 1993;269(18):2386-2391.
- Folstein MF, Folstein SE, McHugh PR. "Mini-mental state": a practical method for grading the cognitive state of patients for the clinician. *J Psychiatr Res*. 1975;12:189-198.
- Rovner BW, Folstein MF. Mini-mental state exam in clinical practice. *Hosp Pract*. 1987;22(1A):99, 103, 106, 110.
- Tombaugh TN, McIntyre NJ. The mini-mental state examination: a comprehensive review. *J Am Geriatr Soc*. 1992;40(9):922-935.
