# Supplementary material for: Preliminary report of Iranian Registry of Alzheimer's disease in Tehran province: A cross‐sectional study in Iran
Source: Health Sci Rep. 2022 Nov 22;5(6):e952. doi: 10.1002/hsr2.952 (PMC9682202; doi:10.1002/hsr2.952)
Supplement: Supplementary file 2 — Supporting information. [file HSR2-5-e952-s001.pdf]

Geriatric Depression Scale (Short Form)

Patient's Name: ______________________________ Date: ______________

Instructions: Choose the best answer for how you felt over the past week. Note: when asking the patient to complete the form, provide the self-rated form (included on the following page).

No.	Question	Answer	Score
1.	Are you basically satisfied with your life?	YES / ***NO***	
2.	Have you dropped many of your activities and interests?	***YES*** / NO	
3.	Do you feel that your life is empty?	***YES*** / NO	
4.	Do you often get bored?	***YES*** / NO	
5.	Are you in good spirits most of the time?	YES / ***NO***	
6.	Are you afraid that something bad is going to happen to you?	***YES*** / NO	
7.	Do you feel happy most of the time?	YES / ***NO***	
8.	Do you often feel helpless?	***YES*** / NO	
9.	Do you prefer to stay at home, rather than going out and doing new things?	***YES*** / NO	
10.	Do you feel you have more problems with memory than most people?	***YES*** / NO	
11.	Do you think it is wonderful to be alive?	YES / ***NO***	
12.	Do you feel pretty worthless the way you are now?	***YES*** / NO	
13.	Do you feel full of energy?	YES / ***NO***	
14.	Do you feel that your situation is hopeless?	***YES*** / NO	
15.	Do you think that most people are better off than you are?	***YES*** / NO	
		TOTAL	

(Sheikh & Yesavage, 1986)

Scoring:

Answers indicating depression are in bold and italicized; score one point for each one selected. A score of 0 to 5 is normal. A score greater than 5 suggests depression.

Sources:

- Sheikh JI, Yesavage JA. Geriatric Depression Scale (GDS): recent evidence and development of a shorter version. *Clin Gerontol*. 1986 June;5(1/2):165-173.
- Yesavage JA. Geriatric Depression Scale. *Psychopharmacol Bull*. 1988;24(4):709-711.
- Yesavage JA, Brink TL, Rose TL, et al. Development and validation of a geriatric depression screening scale: a preliminary report. *J Psychiatr Res*. 1982-83;17(1):37-49.

Geriatric Depression Scale (Short Form)
Self-Rated Version

Patient's Name: ____ Date: ___________________

***Instructions**: Choose the best answer for how you felt over the past week.*

No.	Question	Answer	Score
1.	Are you basically satisfied with your life?	YES / NO	
2.	Have you dropped many of your activities and interests?	YES / NO	
3.	Do you feel that your life is empty?	YES / NO	
4.	Do you often get bored?	YES / NO	
5.	Are you in good spirits most of the time?	YES / NO	
6.	Are you afraid that something bad is going to happen to you?	YES / NO	
7.	Do you feel happy most of the time?	YES / NO	
8.	Do you often feel helpless?	YES / NO	
9.	Do you prefer to stay at home, rather than going out and doing new things?	YES / NO	
10.	Do you feel you have more problems with memory than most people?	YES / NO	
11.	Do you think it is wonderful to be alive?	YES / NO	
12.	Do you feel pretty worthless the way you are now?	YES / NO	
13.	Do you feel full of energy?	YES / NO	
14.	Do you feel that your situation is hopeless?	YES / NO	
15.	Do you think that most people are better off than you are?	YES / NO	
		TOTAL	

(Sheikh & Yesavage, 1986)
